# Primary pulmonary pleomorphic liposarcoma found with a massive hemothorax

**DOI:** 10.1186/s44215-022-00025-y

**Published:** 2023-04-03

**Authors:** Fumi Ohsawa, Natsumi Matsuura, Kazuki Numajiri, Hitoshi Igai, Mitsuhiro Kamiyoshihara

**Affiliations:** Department of General Thoracic Surgery, Japanese Red Cross Maebashi Hospital, 389-1 Asakura-Machi, Maebashi, Gunma 371-0811 Japan

**Keywords:** Pleomorphic liposarcoma, Pulmonary, Hemothorax

## Abstract

**Background:**

Pleomorphic liposarcoma (PLPS) is a rare and aggressive subtype of liposarcoma. Here we report the case of a 45-year-old man with PLPS in the lung, which was accompanied by a massive hemothorax.

**Case presentation:**

The patient presented to hospital with chest pain. A chest x-ray showed massive right pleural effusion. We drained the right chest cavity of 1 L of blood and enhanced computed tomography (CT) revealed a massive right hemothorax and a 78 × 70 mm mass in the right lower lung lobe. The tumor was ruptured and bleeding, so we performed right lower lobe resection. Pathological investigations revealed a PLPS. One month after surgery, positron emission tomography-CT showed considerable pleural dissemination in the right lung field. The patient died 3 months after surgery.

**Conclusions:**

PLPS of the lung is rare, but requires attention because it can cause tumor rupture and hemothorax due to rapid tumor growth, and which may make complete resection of tumor difficult or necessitate emergency surgery.

## Background

Liposarcoma, a common subtype of soft-tissue sarcoma, accounts for 20% of all sarcomas. Pleomorphic liposarcoma (PLPS) is the least common but most aggressive subtype of liposarcoma [1]. PLPS arises most often in the extremities, with the lower limb being most commonly affected. Thus, it is rare for PLPS to arise in the lung and be found with a hemothorax. Here, we report a case of PLPS in the lung found with massive hemothorax.

## Case presentation

**Fig. 1 a:**
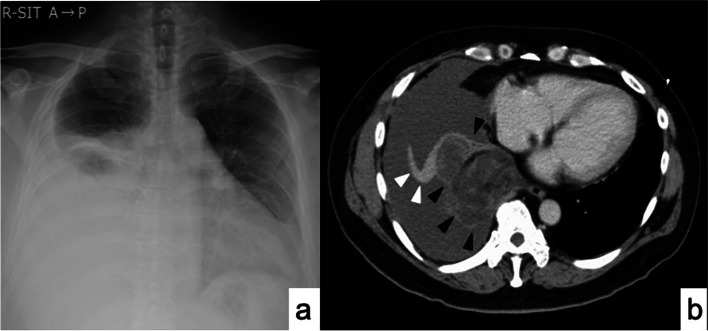
Chest x-ray showing a massive right pleural effusion. **b**. Enhanced chest CT showing a massive right hemothorax and a 78 × 70 mm mass in the right lower lung lobe (▲). The contrast agent (△) had leaked into the thoracic cavity around the tumor

A 45-year-old male visited a local hospital with chest pain and breathing shortness of breath. A chest x-ray showed a massive right pleural effusion (Fig. 1a). Chest x-rays taken 1 and 6 months earlier showed no obvious abnormalities. He then was transported to our hospital. We drained the right chest cavity of 1 L of blood and clamped the drainage tube. Enhanced chest computed tomography (CT) scan revealed a massive right hemothorax and a 78 × 70 mm mass in the right lower lung lobe (Fig. 1b). Extravasation was seen on CT, so an emergency operation was performed. The patient had a history of myocardial infraction, heart failure, and atrial fibrillation, and was taking rivaroxaban and clopidogrel sulfate.

**Fig. 2 a:**
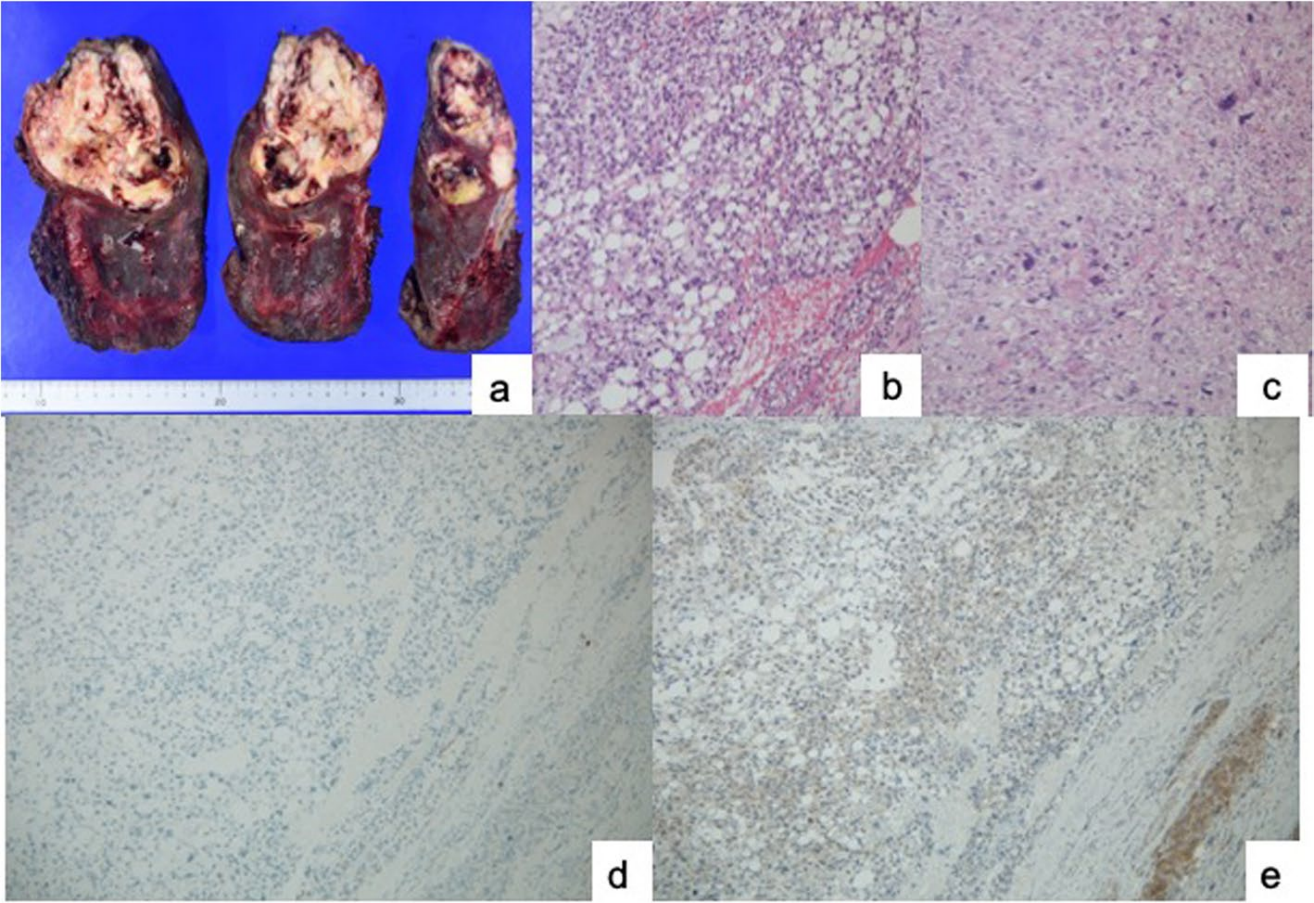
The lower lobe tumor was white, soft, and lobular; had a clear border (8.8 × 7.2 cm); and the contents had split. **b**. Hematoxylin and eosin-stained image showing that the tumor cells had unusual nuclei and increased pleomorphic cytoplasm with broad necrosis. There were numerous lipid droplets within the tumor cells, which were mixed with nuclear lipoblasts. This field contains highly differentiated findings. **c**. This field contains dedifferentiated findings. **d**. Negative immunohistochemistry for MDM2. **e**. Negative immunohistochemistry for CDK4

Surgery was performed via a right posterolateral thoracotomy. First, an intrathoracic hematoma was removed. The tumor of the lower lobe was ruptured and bleeding. Therefore, we diagnosed the hemothorax as resulting from a ruptured lung tumor. While visualization was poor due to the hemorrhage, no obvious pleural seeding was observed. The tumor was large and was inside the lung. A lower lobectomy was necessary for hemostasis. Although the tumor was adherent to the esophagus, there was no gross tumor invasion, and dissection was possible. We removed the tumor grossly and removed the right lower lobe (Fig. 2a). The surgery was an emergency and performed with the highest priority on controlling the bleeding. There was a possibility of microscopic residual tumor since the tumor had ruptured. The volume of bleeding was 2600 mL. The operating time was 175 min. On the second postoperative day, the drainage tube was removed. Arrhythmia developed postoperatively, requiring treatment with cardiologists for 20 days. The patient was discharged on postoperative day 21.

The tumor cells were irregular with unusual nuclei and increased with broad necrosis. There were numerous lipid droplets within the tumor cells and mixed nuclear lipoblasts (Fig. 2b). The mitotic index was 5–6 mitoses per 10 high power fields. Immunohistochemical staining of the tumor indicated highly focal S 100 protein, positive RB protein, and negative MDM2 and CDK4. Pathological investigations revealed a pleomorphic liposarcoma. The right lower lobe near the tumor was adhered to the esophagus and mediastinum, and tumor cell infiltration was seen near the perforation, but there were no findings suggesting tumor initiation from the esophagus or mediastinum.

**Fig. 3 a-b:**
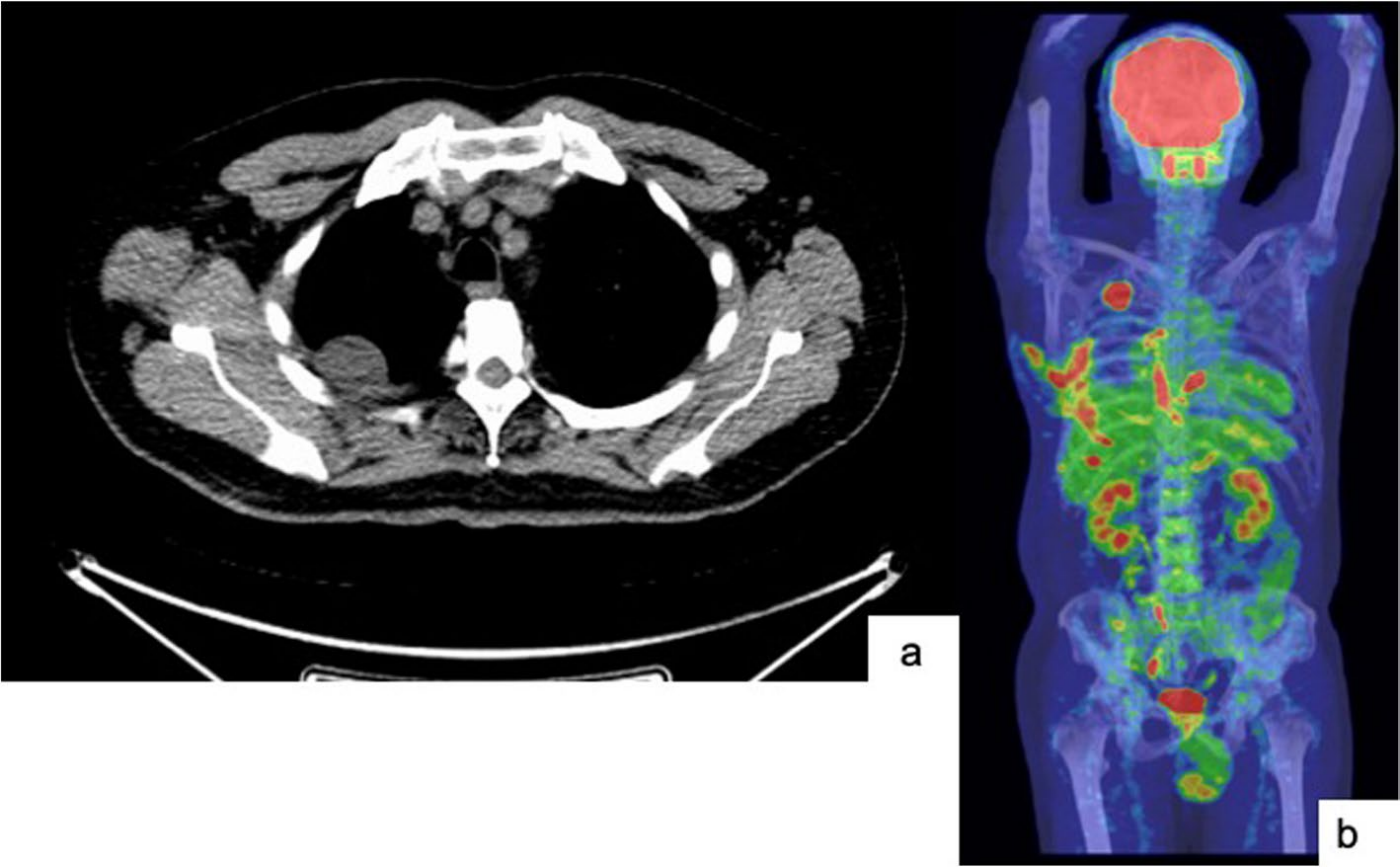
PET-CT at 1 month postoperatively shows considerable pleural dissemination in the right lung field. There was no abnormal uptake in the extremities

One month after surgery, positron emission tomography (PET)-CT showed considerable pleural dissemination in the right lung field (Fig. 2c). We transferred the patient to the soft-tissue-tumor outpatient department of an oncology hospital. After starting chemotherapy with Adriamycin, the patient died 3 months after surgery due to respiratory failure (Fig. 3a-b).

## Discussion and conclusions

Liposarcomas comprise a heterogeneous class of soft-tissue sarcomas with adipocytic differentiation and are among the most common malignancies encountered in soft-tissue pathology [2]. From a histopathological point of view, liposarcomas can be divided into five categories based on the 1994 World Health Organization guidelines. These include well-differentiated (WD; including the lipoma-like, sclerosing, and inflammatory subtypes), myxoid, round-cell (poorly differentiated myxoid), pleomorphic, and dedifferentiated. However, recent identification of morphological features and cytogenetic data indicate three principal groups: atypical lipomatous tumor/WD liposarcoma (ALT/WDLPS) and dedifferentiated liposarcoma (DDLPS); myxoid liposarcoma (MLPS); and PLPS [3]. PLPS is a rare subtype and accounts for 5–10% of all liposarcomas [4]. It has the highest malignancy grade, with high invasion, metastasis, and recurrence. Clinicopathologic and prognostic studies of PLPS have reported recurrence rates of 29–45% and indicated that 35–50% of patients died from the tumor [5, 6]. PLPS mostly appears in middle-aged and older patients (median age range, 54–70 years) and is slightly more common in men than women [2, 3].

PLPS tends to arise in the extremities, most commonly in the lower limb. Although most frequently occurring in deep soft tissues, around 10–20% of cases may be superficially located in the subcutis or, more rarely, the dermis. Other less common sites include the trunk, retroperitoneum, and spermatic cord [2, 7]. PLPS is highly metastatic, and the most common site is the lung followed by the liver, bones, and pancreas [3]. Primary intrathoracic liposarcoma is extremely rare, representing 2.7% of all cases, and can occur in the lung, mediastinum, or pleura [7]. In our case, we performed an emergency operation, so the preoperative examination was deficient. When we performed a PET scan after the operation to find the original lesion genesis, we detected dissemination in the right thoracic cavity, but found no other original lesion. Therefore, we considered this case to be primary pulmonary PLPS. In addition, we could find no other published reports of PLPS with a hemothorax. Thus, our case appears to be extremely rare.

PLPS tends to remain silent clinically silent until large enough to displace adjacent structures [8]. In our case, no tumor was seen on x-ray months before the tumor rupture. The tumor may have grown rapidly. Lin et al. reported a rapidly growing pleural liposarcoma at 2 months. [8] If the growth rate of our case was similar, it would have been difficult to find the tumor when it was still too small to rupture, even if the patient had arrived with other complaints.

Surgery, especially radical resection, is the main treatment for PLPS. However, the local recurrence rate is very high [1, 9]. In addition, the curative effect of chemotherapy on PLPS is controversial. Some reports suggest that conventional chemotherapy is not beneficial for sarcoma patients, and there are still no standardized treatment approaches reported for PLPS [10]. Radiotherapy has shown some value as an adjuvant treatment for MLPS [7, 11]. However, the data are limited for MLPS and nonexistent for PLPS. Thus, at present, radical surgical resection is the best and main treatment for PLPS, with chemotherapy and radiotherapy in multimodality treatment strategies still controversial [3]. In our case, because the tumor was ruptured and had spread to the esophagus, we were not able to do a complete resection during the emergency surgery. We introduced the patient to soft-tissue-tumor specialists, but treatment did not progress rapidly enough. Although very rare, PLPS may present as a pulmonary tumor. If the tumor ruptures in the thorax, emergency surgery may be necessary to treat the resulting hemothorax. In that case, it is difficult to do a complete resection. Therefore, clinicians should recognize that a rapidly growing lung tumor may be PLPS.

PLPS of the lung is a rare, high-grade malignancy with high recurrence, a poor prognosis, and a highly controversial treatment approach. Clinicians should recognize that a rapidly growing lung tumor may be PLPS and result in tumor rupture, which may complicate complete resection of the tumor and require emergency surgery for hemothorax.

## Data Availability

Not applicable.

## Abbreviations

PLPS: Pleomorphic liposarcoma
CT: Computed tomography
PET: Positron emission tomography
WD: Well-differentiated
ALT/WDLPS: Atypical lipomatous tumor/WD liposarcoma
DDLPS: Dedifferentiated liposarcoma
MLPS: Myxoid liposarcoma
